# Examination of mental health, sociodemographic and behavioral factors associated with vigorous physical activity among medical students

**DOI:** 10.3389/fspor.2026.1870798

**Published:** 2026-07-28

**Authors:** Konstantinos Stratakis, Jovana Todorović, Momcilo Mirkovic, Dejan Nešić, Zorica Terzić-Šupić

**Affiliations:** 1 Faculty of Medicine, University of Belgrade, Belgrade, Serbia; 2 Faculty of Medicine, Institute of Social Medicine, University of Belgrade, Belgrade, Serbia; 3 Faculty of Medicine, University of Pristina-Kosovska Mitrovica, Kosovska Mitrovica, Serbia; 4 Faculty of Medicine, Institute for Medical Physiology, University of Belgrade, Belgrade, Serbia

**Keywords:** depression, IPAQ-SF, medical students, mental health, PHQ-9, Serbia, vigorous physical activity

## Abstract

**Introduction:**

Although physical activity (PA) is a multidimensional behavior, most research has focused on total PA volume, meeting PA recommendations, or absolute vigorous physical activity (VPA), rather than on how VPA is distributed within total PA. Therefore, the proportion of VPA within total PA remains an underexplored feature of activity patterns. This exploratory analysis examined whether depressive symptoms and selected sociodemographic, behavioral, health and study-related characteristics were associated with different proportions of VPA within total PA among sufficiently active medical students.

**Methods:**

This was a secondary analysis of data from a cross-sectional study that included 656 sufficiently active fifth-year medical students from five universities in Serbia. PA was assessed using the International Physical Activity Questionnaire (IPAQ-SF), and depressive symptoms were measured with the Patient Health Questionnaire-9 (PHQ-9). Participants were categorized based on the proportion of VPA within total PA into three groups: no VPA contribution, low-to-moderate VPA contribution, and higher VPA contribution, corresponding to 0%, 0.01%–30%, and >30% VPA within total PA.

**Results:**

In pairwise multivariable logistic regression analyses, higher PHQ-9 scores were associated with lower odds of belonging to the 0.01%–30% VPA group than to the 0% VPA group, whereas no significant association was observed for the >30% VPA group. VPA categories were also associated with age, sex, meal frequency, alcohol use, perceived social support, and total energy expenditure.

**Conclusions:**

These findings suggest that, among sufficiently active medical students, depressive symptoms and several sociodemographic, behavioral and health characteristics are associated with differences in VPA distribution within total PA. The association with depressive symptoms was limited to the comparison between no VPA and low-to-moderate VPA proportion. Future longitudinal and experimental studies are needed to clarify these associations beyond the limitations of a cross-sectional self-reported design.

## 1 Introduction

Depression is one of the most common mental health issues worldwide and remains a major contributor to disability and reduced quality of life (1, 2). University students appear to be particularly vulnerable, given the academic, financial, and psychosocial pressures characteristic of this period of life (3). Medical students may be at even greater risk due to intensive academic demands, prolonged study periods, and elevated perceived stress, factors that have been consistently linked to depressive symptoms in this population (4).

Physical activity (PA) has been widely studied in relation to mental health and is recognized as a promising behavioral factor associated with lower depressive symptoms and reduced depression risk (5, 6). However, PA is a multidimensional behavior, and most studies have focused primarily on total PA volume, without adequately considering how activity is distributed across intensity levels. This distinction may be important, as the World Health Organization recommends that adults accumulate PA through different combinations of moderate- and vigorous-intensity activity, allowing substantial variability in intensity profiles even among individuals who meet the same overall PA recommendations (7). This issue may be particularly relevant in medical students, whose academic workload, time constraints, and health-related education may shape lifestyle behaviors and the way recommended PA is accumulated.

In recent years, interest has grown in the idea that not only total PA volume, but also the proportion of vigorous physical activity (VPA) within total PA, may be relevant for health outcomes. The proportion of VPA is not another measure of absolute VPA duration. It describes how much of an individual's overall activity profile is composed of VPA. This distinction is important because two individuals may accumulate similar amounts of total PA, while differing substantially in whether their activity is mainly walking, moderate-intensity activity, or VPA. Existing epidemiological studies have linked higher proportions of VPA with favorable physical health outcomes, including lower all-cause and cardiovascular mortality (8, 9). Nevertheless, the proportion of VPA within total PA remains a relatively underexplored feature of activity patterns, particularly in young adult populations and in relation to mental health, sociodemographic and behavioral characteristics.

The possible relevance of VPA for mental health is supported by several biological and psychological mechanisms. VPA is associated with greater lactate production, which has been linked to peripheral and central increases in brain-derived neurotrophic factor (BDNF), a molecule implicated in neuroplasticity (10). High-intensity exercise may also elicit stronger opioidergic responses and may influence regulation of the hypothalamic–pituitary–adrenal axis, thereby affecting mood and stress reactivity (11, 12). From a psychological perspective, VPA may be associated with enhanced self-efficacy, mastery, and short-term mood improvements (13, 14). At the same time, these mechanisms do not imply a direct or causal relationship within observational studies; rather, they support considering depressive symptoms as a relevant psychological correlate when examining VPA distribution.

Empirical findings on the relationship between VPA and depressive symptoms, however, remain inconsistent. An accelerometer-based analysis of approximately 90,000 adults from the UK Biobank found that, after accounting for total PA volume, higher proportions of VPA were not associated with further reductions in depressive symptoms (15). In contrast, analyses of NHANES data reported that higher PA intensity was associated with lower depressive symptom risk, particularly in certain subgroups (16). These mixed findings suggest that depressive symptoms may be relevant to VPA distribution, but that this relationship is likely context-dependent and not yet well defined.

Importantly, VPA patterns do not develop in isolation. PA behavior in university students is shaped by a broader combination of sociodemographic, lifestyle, and psychosocial factors. Recent evidence indicates that activity and sedentary behavior in university students vary according to sex, age, field of study, and other contextual characteristics (17). Among medical students from the Western Balkans, regular PA has been associated with male sex, higher household income, and lower overweight/obesity prevalence (18), while motives and barriers for regular PA vary according to gender, BMI, income, smoking, and other lifestyle-related factors (19). In addition, VPA in college populations has been linked not only to mental health and perceived stress, but also to socializing, suggesting that VPA may reflect broader behavioral and psychosocial patterns (20).

Overall, the available evidence suggests that depressive symptoms may be a relevant mental health characteristic to examine in relation to VPA and that the proportion of VPA within total PA should be considered an underexplored behavioral feature that may vary according to a broader set of sociodemographic and behavioral characteristics. This may be particularly relevant in sufficiently active populations, where the key distinction is not whether people are active or inactive, but how activity is distributed across intensity levels among those already meeting recommended PA levels.

Therefore, the present study was conducted as an exploratory analysis to examine whether depressive symptoms and selected sociodemographic, behavioral, health and study-related characteristics were associated with differences in the proportion of VPA within total PA among sufficiently active medical students. The specific contribution of this study is its focus on VPA distribution within total PA, rather than total PA volume or absolute VPA alone, in a multi-university sample of students. The analysis was restricted to participants meeting the WHO minimum recommendations for PA because the study aimed to compare how activity was distributed across intensity levels among students who had already achieved the same minimum PA standard. Students in the 0% VPA group could still meet recommendations through walking and/or moderate-intensity PA, whereas students in the other groups met recommendations through different combinations of moderate- and vigorous-intensity PA. Accordingly, the findings should be interpreted as associations within a sufficiently active medical-student population and should not be generalized to inactive students or broader populations.

## 2 Materials and methods

### 2.1 Study design and participants

This study was a secondary analysis of data collected through a cross-sectional survey conducted during the 2023/2024 academic year among fifth-year medical students at five universities in Serbia who attended Social Medicine classes (Belgrade, Novi Sad, Kragujevac, Niš, and Kosovska Mitrovica). Data collection was conducted during mandatory Social Medicine practical sessions during the winter semester. Eligible participants were students who were present in class at the time of data collection. Students were informed about the study and invited to complete an anonymous, paper-based, self-administered questionnaire. Before questionnaire completion, participants received standardized information about the study purpose, voluntary participation, anonymity, and the procedure for completing the questionnaire. Participation was voluntary, and questionnaire completion implied informed consent. A total of 730 students completed the survey (response rate: 68.9%). For the present secondary analysis, participants were included if they completed the questionnaire and met the WHO minimum recommendations for PA for health benefits. In this study, “sufficiently active” referred to participants who met the WHO minimum adult PA recommendation of at least 150 min of moderate-intensity PA per week, at least 75 min of VPA per week, or an equivalent combination of moderate- and vigorous-intensity activity. Participants who did not meet these recommendations were excluded, resulting in a final analytic sample of 656 students. The overall response rate was available, but the exact number of students invited from each university was not retained in the analytic dataset; therefore, university-specific response rates could not be calculated. Although students were recruited from five universities, the present analysis was not designed to compare universities. The aim was to examine correlates of VPA proportion categories in the pooled sample of sufficiently active medical students. Restricting the sample to sufficiently active individuals allowed comparison of PA intensity-distribution patterns among students who had already met the same minimum WHO PA standard. Students classified in the 0% VPA group were sufficiently active through walking and/or moderate-intensity PA, whereas students in the other groups accumulated PA through different combinations of moderate- and vigorous-intensity activity. The restriction was therefore used to define the target population for this exploratory analysis rather than to compare sufficiently active and inactive students. The survey used in this study has also contributed to two related analyses (21, 22). One published study examined total PA categories and factors associated with PA in a broader pre-/post-COVID comparison of medical students (21). A second published study examined the dose-response association between total PA volume and anxiety symptoms across two academic years in medical students from Serbia (22). These studies addressed different research questions and used different PA exposure definitions and mental-health outcomes, and did not examine VPA proportion within total PA as the primary outcome.

### 2.2 Measures

PA was assessed using the short form of the International Physical Activity Questionnaire (IPAQ-SF), a widely used self-report instrument developed for cross-national monitoring of physical activity and inactivity (23). Although IPAQ-SF has been evaluated internationally, validity estimates vary across populations and self-reported PA may be affected by recall error and misclassification of activity intensity (23–25). Serbian reliability evidence is available for IPAQ in older adults; however, this does not establish validity or measurement equivalence of IPAQ-SF in Serbian medical students (26). Total PA was expressed as metabolic equivalent task minutes per week (MET-min/week) and calculated according to the guidelines for calculating energy expenditure based on IPAQ-SF (23). To examine the relative contribution of VPA within total PA, VPA MET-min/week was divided by total PA MET-min/week, including walking, moderate-intensity PA, and vigorous-intensity PA, and multiplied by 100. Thus, the VPA-proportion variable reflects the relative contribution of VPA to total self-reported PA volume rather than an independent measure of absolute VPA duration. Participants were categorized into three groups: no VPA contribution, low-to-moderate VPA contribution, and higher VPA contribution, corresponding to 0%, 0.01%–30%, and >30% VPA within total PA, respectively. These thresholds were based on prior epidemiological studies examining the distribution of VPA within total PA (8, 9). The conceptual rationale was to distinguish between students whose activity profile included no VPA, students for whom VPA represented a limited component of total PA, and students for whom VPA represented a more pronounced component of total PA. Depressive symptoms were assessed using the Patient Health Questionnaire-9 (PHQ-9), a nine-item self-report instrument assessing depressive symptom severity over the previous two weeks (27). Total scores range from 0 to 27, with higher scores indicating greater symptom severity. The PHQ-9 was analyzed as a continuous self-report measure of depressive symptom severity and was not used to establish a clinical diagnosis of depressive disorder. Serbian general-population evidence regarding PHQ-9 screening performance is also available, although this does not constitute validation in Serbian medical students (28). The questionnaire also collected information sociodemographic and lifestyle characteristics considered theoretically relevant to PA patterns, including university affiliation, place of residence, sex, age, relationship status, self-rated financial status, grade point average (GPA), body mass index (BMI) calculated from self-reported height and weight, family relationships, and self-rated health. Lifestyle-related variables comprised tobacco use, alcohol consumption during the past 12 months, binge drinking, cannabis use, time spent on social media and video games, number of meals per day, perceived social support assessed using the Multidimensional Scale of Perceived Social Support (MSPSS) (29, 31), and study engagement assessed using the study-engagement measure applied by Salmela-Aro and Read among Finnish higher-education students (32). The MSPSS was originally developed as a multidimensional measure of perceived support from family, friends, and significant others (30). It has demonstrated acceptable psychometric properties across diverse populations, including a Serbian psychometric evaluation that supported internal consistency and the expected three-factor structure in a Serbian LGBT sample; however, this evidence does not establish measurement equivalence in Serbian medical students (29, 31). The study-engagement measure was derived from the instrument applied by Salmela-Aro and Read among Finnish higher-education students; however, formal psychometric validation of this measure in Serbian medical students has not been established (32). All questionnaires were administered in Serbian. For IPAQ, the available Serbian version had previously undergone independent forward translation, reconciliation of translation differences, and back-translation. Serbian-language forms of the PHQ-9, MSPSS, and study-engagement measure were also used. However, no additional translation or cultural-adaptation procedures were undertaken as part of the present study, and instrument-specific psychometric estimates were not available for the current sample.

### 2.3 Statistical analyses

Descriptive statistics were conducted for all study variables. Differences between categorical variables were examined using the chi-square test. For continuous variables, one-way analysis of variance (ANOVA) was applied when assumptions of normality were met, and the Kruskal–Wallis test was used for non-normally distributed variables. Normality was assessed using the Kolmogorov–Smirnov test. For all variables *post hoc* pairwise comparisons were performed to identify which specific groups differed. Pairwise comparisons for categorical variables were examined using *post hoc* chi-square tests with adjustment for multiple comparisons. For continuous variables, *post hoc* pairwise comparisons were conducted using Tukey's test after ANOVA or Dunn–Bonferroni tests after Kruskal–Wallis tests, depending on distribution. These *post hoc* comparisons were used to improve the interpretability of the descriptive analyses and were not used as criteria for selecting variables for the multivariable models. The multivariable analyses were designed to examine whether depressive symptoms and selected sociodemographic and lifestyle characteristics were associated with different VPA proportion categories. Accordingly, VPA proportion category was treated as the outcome variable, while depressive symptoms and the other selected characteristics were examined as independent variables in relation to this outcome. This approach was chosen because the proportion of VPA within total PA represents an underexplored behavioral feature of PA patterns, and the present analysis was intended to identify factors associated with it, rather than estimating a causal effect of VPA proportion or an effect independent of total PA. Candidate variables for multivariable analyses were identified based on their theoretical relevance to PA behavior. To preserve model parsimony in relation to the sample size, the number of candidate predictors, and the smaller size of the 0.01%–30% VPA group, bivariate analyses were additionally used to inform final model specification. Thus, theoretically relevant variables that showed an overall association with the three-category VPA proportion outcome in univariate analyses (*p* < 0.05) were entered into the multivariable models. The overall univariate tests were used to identify candidate variables associated with VPA proportion category, while the subsequent pairwise logistic regression models were used to examine the predefined adjusted contrasts between VPA proportion groups. This approach was adopted in keeping with the exploratory nature of the study and should not be interpreted as a fully adjusted causal modeling strategy. Pairwise binary logistic regression models were then constructed for the following predefined comparisons: (1) >30% VPA vs. 0% VPA, (2) 0.01%–30% VPA vs. 0% VPA, and (3) >30% VPA vs. 0.01%–30% VPA. This pairwise approach was chosen to allow direct interpretation of differences between clinically and behaviorally distinct VPA proportion categories. Results were expressed as odds ratios (ORs) with 95% confidence intervals (CIs). Multicollinearity was assessed using the Variance Inflation Factor (VIF), with values below 5 considered acceptable. Goodness of fit was assessed via the Nagelkerke R^2^. All statistical analyses were performed using IBM SPSS Statistics version 22.0.

## 3 Results

A total of 224 students (34.1%) were in the 0% VPA in total PA group, 108 students (16.5%) were in the 0.01%–30% VPA in total PA group, and 324 students (49.4%) were in the >30% VPA in total PA group. Mean energy expenditure in the sample was 3,128.52 ± 2,253.57 MET-minutes/week, while the mean PHQ-9 score was 5.49 ± 4.64**.** A total of 102 students (16.2%) had a PHQ-9 score of ≥10, indicating a clinically significant score. There were significant overall differences across the three VPA proportion groups in sex, self-rated financial status, alcohol use in the past year, binge drinking in the past month, number of meals per day, perceived social support, age, BMI, PHQ-9 score, and energy expenditure. Sociodemographic characteristics are presented in Table 1, behavioral characteristics are presented in Table 2, and health and study-related characteristics are presented in Table 3.

**Table 1:** Sociodemographic characteristics of sufficiently active medical students according to the proportion of VPA within total PA, *n* = 656.

Characteristics	0% VPA in total PA	0.01%–30% VPA in total PA	>30% VPA in total PA	Overall *p*-value	Post hoc *p*-value: 0% VPA in total PA vs. 0.01%–30% VPA in total PA	Post hoc *p*-value: 0% VPA in total PA vs. >30% VPA in total PA	Post hoc *p*-value: 0.01%–30% VPA in total PA vs. >30% VPA in total PA
Residence				0.701	0.559	0.782	0.400
Urban	194 (87.4)	91 (85.0)	276 (88.2)				
Rural	28 (12.6)	16 (15.0)	37 (11.8)				
Sex				0.001	0.004	<0.001	0.103
Male	46 (20.5)	37 (35.2)	143 (44.3)				
Female	178 (79.5)	68 (64.8)	180 (55.7)				
Self-rated financial status				0.018	0.169	0.020	0.074
Poor	8 (3.6)	7 (6.6)	7 (2.2)				
Average	106 (47.3)	40 (37.7)	119 (36.8)				
Good	110 (49.1)	59 (55.7)	197 (61.0)				
Family relationships				0.067	0.320	0.047	0.157
Poor	7 (3.2)	7 (6.5)	11 (3.4)				
Average	44 (19.8)	18 (16.7)	39 (12.1)				
Good	171 (77.0)	83 (76.9)	273 (84.5)				
Relationship status				0.055	0.618	0.043	0.035
Single	104 (46.4)	47 (44.8)	179 (55.2)				
In a relationship	120 (53.6)	58 (55.2)	145 (44.8)				

Values are presented as *n* (%), with percentages calculated within each VPA proportion group. Overall *p*-values indicate differences across the three VPA proportion groups and were examined using the chi-square test. *post hoc p*-values indicate pairwise comparisons between VPA proportion groups. PA, physical activity; VPA, vigorous physical activity.

**Table 2:** Behavioral characteristics of sufficiently active medical students according to the proportion of VPA within total PA, *n* = 656.

Characteristics	0% VPA in total PA	0.01%–30% VPA in total PA	>30% VPA in total PA	Overall *p*-value	Post hoc *p*-value: 0% VPA in total PA vs. 0.01%–30% VPA in total PA	Post hoc *p*-value: 0% VPA in total PA vs. >30% VPA in total PA	Post hoc *p*-value: 0.01%–30% VPA in total PA vs. >30% VPA in total PA
Cannabis use				0.510	0.314	0.321	0.786
Yes	26 (11.8)	16 (15.8)	47 (14.7)				
No	195 (88.2)	85 (84.2)	272 (85.3)				
Smoking				0.239	0.888	0.144	0.187
Yes	55 (27.0)	28 (27.7)	64 (21.3)				
No	149 (73.0)	73 (72.3)	236 (78.7)				
Alcohol use in the past year				0.004	0.087	0.078	<0.001
Yes	185 (83.0)	81 (75.0)	286 (88.3)				
No	38 (17.0)	27 (25.0)	38 (11.7)				
Binge drinking in the past month				0.003	0.708	0.007	0.012
Yes	54 (25.5)	35 (34.3)	123 (39.7)				
No	158 (74.5)	67 (65.7)	187 (60.3)				

Values are presented as *n* (%), with percentages calculated within each VPA proportion group. Overall *p*-values indicate differences across the three VPA proportion groups and were examined using the chi-square test. *post hoc p*-values indicate pairwise comparisons between VPA proportion groups. PA, physical activity; VPA, vigorous physical activity.

**Table 3:** Health and study-related characteristics of sufficiently active medical students according to the proportion of VPA within total PA, *n* = 656.

Characteristics	0% VPA in total PA	0.01%–30% VPA in total PA	>30% VPA in total PA	Overall *p*-value	Post hoc *p*-value: 0% VPA in total PA vs. 0.01%–30% VPA in total PA	Post hoc *p*-value: 0% VPA in total PA vs. >30% VPA in total PA	Post hoc *p*-value: 0.01%–30% VPA in total PA vs. >30% VPA in total PA
GPA, X ± SD	8.87 ± 0.73	8.91 ± 0.72	8.91 ± 1.03	0.700	0.910	0.451	0.562
Time spent on social media, hours/day, X ± SD	3.15 ± 1.65	3.30 ± 1.86	2.89 ± 1.88	0.170	0.844	0.084	0.212
Number of meals per day, X ± SD	2.80 ± 0.78	2.81 ± 0.70	3.33 ± 0.96	0.001	0.407	<0.001	0.002
Social support, X ± SD	6.06 ± 0.74	6.37 ± 0.48	6.15 ± 0.95	0.011	0.654	0.640	0.020
Study engagement, X ± SD	32.16 ± 6.60	34.03 ± 7.15	32.80 ± 8.96	0.238	0.275	0.112	0.916
Age in years, X ± SD	24.29 ± 1.18	24.37 ± 1.75	24.75 ± 2.10	0.026	0.037	0.517	0.007
BMI in kg/m², X ± SD	22.16 ± 3.84	23.20 ± 3.09	22.91 ± 3.21	0.001	0.173	<0.001	0.054
PHQ-9 score, X ± SD	5.85 ± 4.60	5.25 ± 3.78	5.54 ± 5.06	0.001	0.045	<0.001	0.353
Energy expenditure in MET-minutes/week, X ± SD	2,081.98 ± 1,524.24	3,694.16 ± 2,159.77	3,590.25 ± 2,310.17	0.001	<0.001	<0.001	0.520

Values are presented as mean ± standard deviation. Overall *p*-values indicate differences across the three VPA proportion groups and were examined using one-way ANOVA or Kruskal–Wallis test, depending on distribution. *post hoc p*-values indicate pairwise comparisons between VPA proportion groups. GPA, grade point average; BMI, body mass index; PHQ-9, Patient Health Questionnaire-9; PA, physical activity; VPA, vigorous physical activity.

Pairwise multivariable logistic regression model with more than >30% VPA in total PA compared to 0% of VPA in total PA showed the association of >30% VPA in total PA with female sex (OR: 0.45, 95% CI: 0.26–0.77), number of meals per day (OR: 1.45, 95% CI: 1.12–1.87), age in years (OR:1.23, 95% CI: 1.06–1.44) and energy expenditure in MET-minutes/week (OR:1.01, 95% CI: 1.00–1.01). Multivariable logistic regression model showed that 0.01%–30% VPA in total PA, compared to 0% VPA in total PA, was associated with PHQ-9 score (OR: 0.93, 95% CI: 0.86–0.99) and energy expenditure in MET-minutes/week (OR:1.01, 95% CI: 1.00–1.01). Multivariable logistic regression model with more than >30% VPA in total PA compared to 0.01%–30% of VPA in total PA showed the association of >30% VPA in total PA with alcohol use in the past year (OR: 2.66, 95% CI: 1.33–5.30), number of meals per day (OR: 1.39, 95% CI: 1.05–1.84), social support (OR: 1.45, 95% CI: 1.06–1.99), and age in years (OR: 1.25, 95% CI: 1.04–1.50). The results of the multivariable logistic regression analyses are presented in Table 4.

**Table 4:** Pairwise multivariable logistic regression analyses of factors associated with VPA proportion categories among sufficiently active medical students, *n* = 656.

Characteristics	>30% VPA in total PA vs. 0% VPA in total PA	0.01–30% VPA in total PA vs. 0% VPA in total PA	>30% VPA in total PA vs. 0.01–30% VPA in total PA
Sex			
Male	1.0 reference category	1.0 reference category	1.0 reference category
Female	**0.45** (**0.26–0.77)**	0.56 (0.28–1.11)	0.63 (0.34–1.16)
Self-rated financial status			
Poor	1.0 reference category	1.0 reference category	1.0 reference category
Average	2.12 (0.46–9.75)	0.43 (0.11–1.60)	3.91 (0.94–16.21)
Good	3.22 (0.70–14.85)	0.68 (0.18–2.55)	4.02 (0.99–16.24)
Alcohol use in the past year			
Yes	1.29 (0.69–2.44)	0.61 (0.30–1.23)	2.66 (1.33–5.30)
No	1.0 reference category	1.0 reference category	1.0 reference category
Binge drinking in the past month			
Yes	1.60 (0.98–2.61)	1.43 (0.75–2.74)	1.02 (0.56–1.85)
No	1.0 reference category	1.0 reference category	1.0 reference category
Number of meals per day	**1.45** (**1.12–1.87)**	1.09 (0.79–1.52)	**1.39** (**1.05–1.84)**
Social support	1.24 (0.90–1.71)	0.82 (0.57–1.16)	**1.45** (**1.06–1.99)**
Age in years	**1.23** (**1.06–1.44)**	0.99 (0.80–1.21)	**1.25** (**1.04–1.50)**
BMI in kg/m2	0.99 (0.93–1.06)	1.01 (0.93–1.10)	1.00 (0.92–1.09)
PHQ-9 score	0.97 (0.92–1.01)	**0.93** (**0.86–0.99)**	1.02 (0.96–1.09)
Energy expenditure in MET-minutes/week	1.01 (1.00–1.01)	1.01 (1.00–1.01)	1.0 (.0.99–1.01)
Nagelkerke R square	0.28	0.18	0.14

Values are presented as odds ratios with 95% confidence intervals. Statistically significant associations are shown in bold. The VPA proportion categories were defined as follows: 0% VPA in total PA indicates no VPA contribution; 0.01%–30% VPA in total PA indicates low-to-moderate VPA contribution; and >30% VPA in total PA indicates higher VPA contribution. Percentage-based labels were retained in the table headings to preserve the exact predefined pairwise comparisons used in the regression models. Candidate variables were selected based on theoretical relevance and overall univariate significance across the three VPA proportion groups. Total energy expenditure was entered as MET-minutes/week; therefore, the odds ratio for this variable reflects a one-unit increase in MET-minutes/week. Statistically significant associations are shown in bold. BMI, body mass index; PHQ-9, Patient Health Questionnaire-9; PA, physical activity; VPA, vigorous physical activity.

## 4 Discussion

The current study explored whether depressive symptoms and selected sociodemographic, behavioral, health and study-related characteristics were associated with differences in VPA proportion categories among sufficiently active medical students from five universities in Serbia. Higher PHQ-9 scores were associated with a lower likelihood of belonging to the 0.01%–30% VPA group compared to those reporting no VPA. However, no significant association was observed between depressive symptoms and the >30% VPA category.

Previous research consistently demonstrates that PA is associated with lower depressive symptoms and a reduced risk of depression (5, 6). However, much of this research treats PA as a single construct and does not separate effects by the proportion of VPA within total PA Large-scale investigations, including those by Gebel et al. (8) and Wang et al. (9), have found reduced risks of all-cause and cardiovascular mortality when VPA constitutes at least 30% of total PA (8, 9). In contrast, findings related to mental health outcomes are less consistent (15, 16). For instance, the accelerometer-based analysis of UK Biobank participants indicated that, when total PA volume was controlled, higher proportions of VPA did not further reduce depressive symptoms (15). Conversely, analyses of NHANES data revealed that greater proportions of VPA were associated with a lower likelihood of depressive symptoms, particularly among men and individuals over 45 years of age (16). In our study, each one-point increase in PHQ-9 score was associated with a 7% decrease in the odds of belonging to the 0.01%–30% VPA group compared to the 0% VPA group. Despite the modest magnitude of the association, it remained statistically significant after adjustment for selected sociodemographic, behavioral, health and study-related characteristics and total PA expressed as total MET-minutes/week. Total energy expenditure was included as a covariate to describe the association of VPA-proportion category with overall self-reported PA volume within the selected active population. Because VPA proportion is mathematically derived from total PA, this adjustment should not be interpreted as demonstrating an association independent of total PA volume. Notably, we did not observe significant associations between depressive symptoms and the >30% VPA group compared with either the 0% VPA group or the 0.01%–30% VPA group. These findings suggest that depressive symptoms may be associated with specific differences in VPA distribution, but they do not indicate a consistent association across all VPA proportion categories. The absence of a significant association in the >30% VPA group in the present study is consistent with the broader literature, which does not yet support a uniform relationship between higher VPA proportion and depressive symptoms (15, 16). Because the study design was cross-sectional, the direction of the association cannot be determined, and reverse causality remains a major interpretive limitation. Students experiencing more depressive symptoms may be less likely to engage in vigorous activity due to fatigue, reduced motivation, or lower energy levels; therefore, the observed association should not be interpreted as evidence that VPA distribution directly affects depressive symptoms (33). The absence of a uniform association with depressive symptoms also supports examining VPA proportion in relation to broader sociodemographic, behavioral, health and study-related characteristics. In our analysis, several such characteristics were associated with the proportion of VPA within total PA.

Compared to students who reported no VPA, those in the >30% VPA group were less likely to be female. This finding is consistent with previous research indicating that men typically participate more frequently in VPA and organized sports than women, particularly in university populations (34). In student populations, such differences may partly reflect gendered patterns in sport participation, exercise preferences, access to vigorous recreational activities, and social norms around high-intensity activity. However, the present study did not assess type of activity or motivations for exercise, so these explanations remain speculative. Additionally, age was positively associated with the likelihood of belonging to the >30% VPA group, suggesting that slightly older students in this cohort may engage more often in structured or intentional exercise behaviors. Students in this group also reported a higher number of meals per day. Because the present study did not assess total dietary intake, meal size, dietary composition, or energy balance, this finding should be interpreted only as a behavioral correlate and not as evidence of greater energy intake or greater energy requirements. When comparing the >30% VPA group with the 0.01%–30% VPA group, higher levels of VPA were also linked to alcohol use over the past year and greater perceived social support. Similar patterns have been observed in previous studies among university students, in which participation in sports and VPA often occurs in social settings involving alcohol consumption (35). Noticeably, the present study did not assess the context in which VPA was performed, and this explanation should therefore be interpreted cautiously. The positive association with social support may indicate that engaging in more intensive forms of PA is encouraged by supportive peer networks and shared activity contexts. Taken together, these findings suggest that VPA proportion may reflect a broader pattern of mental health, lifestyle, and sociodemographic characteristics rather than a single isolated behavior. At the same time, not all examined characteristics were independently associated with VPA proportion categories. Variables such as BMI, binge drinking, self-rated financial status, and several pairwise comparisons for sex were not consistently significant in the adjusted models. This is not unexpected, as previous studies in university and medical student populations show that PA patterns are shaped by multiple sociodemographic and lifestyle factors, but that these associations vary across settings, populations, and activity measures (17–19). In the present study, the restriction to sufficiently active fifth-year medical students may also have reduced variability in some characteristics, making weaker associations more difficult to detect. This study has several strengths, including a relatively large multi-university sample, examination of a variety of social and lifestyle characteristics, and a focus on intensity distribution rather than PA volume alone. However, several limitations should be considered when interpreting these findings. First, the cross-sectional design precludes establishing causal relationships. Second, the analysis was restricted to sufficiently active Serbian medical students enrolled in Social Medicine classes during the study period. This restriction was appropriate for the specific aim of comparing PA intensity-distribution profiles within an already active population; however, it limits generalizability to inactive students, students from other disciplines, and young adults outside medical education. Third, all variables were derived from self-reported data, which may introduce measurement error. This limitation is particularly relevant for IPAQ-SF because recall error and misclassification of walking, moderate-intensity PA, or VPA may affect both total PA and the calculated proportion of VPA within total PA. Moreover, formal validation of IPAQ-SF, PHQ-9, MSPSS, and the study-engagement measure in Serbian medical students remains limited. Potential differences in the linguistic and contextual interpretation of questionnaire items may therefore have influenced measurement precision and limit direct comparison with studies using fully validated language-specific instruments. In addition, several potentially relevant variables were not available in the dataset, including sleep quality, academic stress, chronic medical conditions, psychiatric history, and type of sport or exercise. Residual confounding is therefore possible. Finally, the study population consisted solely of sufficiently active fifth-year medical students from Serbia; therefore, the findings should be interpreted within this specific context and may not be generalizable to inactive students, students from other fields, or young adults outside medical education.

## 5 Conclusions

This exploratory analysis suggests that the proportion of VPA within total PA is associated with depressive symptoms and several sociodemographic and lifestyle characteristics among sufficiently active medical students. In particular, depressive symptoms were associated only with the comparison between students reporting no VPA and those reporting low-to-moderate proportions of VPA, while no clear graded relationship was observed across higher VPA categories. These findings indicate that VPA distribution may reflect broader behavioral and psychosocial patterns rather than overall activity volume alone. From a student mental health perspective, the findings suggest that university health promotion programs may benefit from considering not only whether students are physically active, but also how activity is distributed across intensity levels and how these patterns relate to lifestyle and psychosocial characteristics. Given the cross-sectional design and reliance on self-reported measures, the results should be interpreted cautiously. Further longitudinal and experimental research is needed to clarify these associations, examine whether changes in VPA distribution are related to changes in depressive symptoms, and better understand the correlates of VPA distribution in student populations.

## Data Availability

The raw data supporting the conclusions of this article will be made available by the authors, without undue reservation.
